# Using Nonlinear Dynamics and Multivariate Statistics to Analyze EEG Signals of Insomniacs with the Intervention of Superficial Acupuncture

**DOI:** 10.1155/2020/8817843

**Published:** 2020-11-17

**Authors:** Shi-Yi Qi, Dong Lin, Li-Li Lin, Xiao-Zhen Huang, Shen Lin, Yun-Ying Yu, Chuan-Hai Cao, Zhi-Xin Wang

**Affiliations:** ^1^College of Acupuncture, Fujian University of Traditional Chinese Medicine, Fuzhou, Fujian Province, China; ^2^Department of Traditional Chinese Medicine Rehabilitation, Anxi County Hospital, Quanzhou, Fujian Province, China; ^3^Department of Sleep Medicine, Rehabilitation Hospital Affiliated to Fujian University of Traditional Chinese Medicine, Fuzhou, Fujian Province, China; ^4^Department of Neurosurgery and Brain Repair, Morsani College of Medicine, University of South Florida, Tampa, FL, USA; ^5^Department of Internal Medicine, Florida Orthopedic Institute, University of South Florida, Tampa, FL, USA

## Abstract

**Objective:**

As a noninvasive and nonpharmacological therapeutic approach, superficial acupuncture (SA) is a special method of acupuncture. In this study, using nonlinear dynamics and multivariate statistics, we studied the electroencephalography (EEG) of primary insomnia under SA intervention to investigate how brain regions change.

**Method:**

This study included 30 adults with primary insomnia. They underwent superficial acupuncture at the Shangen acupoint. The EEG signals were collected for 10 minutes at each state, including the resting state, the intervention state, and the postintervention state. The data were conducted using nonlinear dynamics (including approximate entropy (ApEn) and correlation dimension (CD)) and multivariate statistics.

**Result:**

The repeated-measures ANOVA results showed that both ApEn and CD values were not significantly different at the three states (*p* > 0.05). The paired *t*-test results showed that the ApEn values of electrodes O2 (the right occipital lobe) at the postintervention state have decreased, compared with the resting state (*p* < 0.05), and no difference was detected in CD (*p* > 0.05). The cluster analysis results of ApEn showed that patients' EEG has changed from the right prefrontal lobe (electrode Fp2) to the right posterior temporal lobe (electrode T6) and finally to the right occipital lobe (electrode O2), before, during, and after the SA intervention. In addition, the factor analysis results of CD revealed that patients' EEG of all brain regions except for the occipital lobes has changed to the frontal lobes and anterior temporal and frontal lobes from pre- to postintervention.

**Conclusion:**

SA activated the corresponding brain regions and reduced the complexity of the brain involved. It is feasible to use nonlinear dynamics analysis and multivariate statistics to examine the effects of SA on the human brain.

## 1. Introduction

Insomnia is one of the most common sleep disorders and can have negative impacts on patients' quality of life; it is also associated with medical morbidity [1, 2]. Approximately a third of the population experience at least a mild form of insomnia, with 6–10% even meeting the diagnostic criteria for insomnia syndrome [3, 4]. Unfortunately, current treatments often produce adverse effects, such as drug dependence, depression, and amnesia [3, 5]. Therefore, it is necessary and crucial to explore nondrug treatments for insomnia [6].

Acupuncture is a treatment that has recently gained more and more attention from researchers as a nonpharmaceutical treatment for insomnia [7, 8]. A number of studies showed that insomnia is associated with hyperarousal in daily life, as well as overactivation of the central nervous system (CNS) and the autonomic nervous system (ANS) [9, 10]. Studies have also demonstrated that acupuncture not only can improve many diseases of the CNS, such as Alzheimer's [11], focal ischemic stroke [12], etc. but also causes autonomic remodeling by improving the balance between the vagus nerve and the sympathetic nervous system [13, 14].

**Figure 1:**
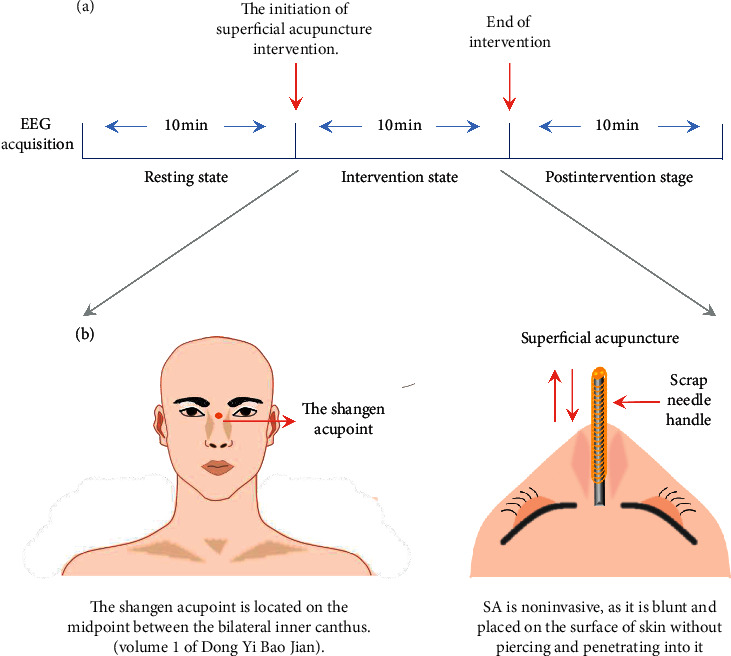
Flow diagram showing the intervention programme. (a) Experiment design of EEG measurement. (b) The location of the Shangen acupoint and introduction of SA.

Superficial acupuncture (SA) (alias shallow acupuncture) is a special form of acupuncture that was first recorded in the Internal Classic of the Yellow Emperor (Huang Di Neijing) in ancient China. SA is noninvasive, as it is blunt and placed on the surface of the skin without piercing and penetrating into it (see Figure 1(b)). Nowadays, it is even smaller and more exquisite. The needle is about 9 cm in length, and the needle handle is about 6 cm in length, which is wound with copper wire wrapping around. The acupuncturist held the needle handle with two right-hand fingers (index finger and middle finger) and scraped the needle handle at a uniform velocity for many minutes with his or her thumb to generate vibration wave at the acupoint. SA is widely used in Fujian province, China, and an increasing number of acupuncturists practice SA therapy to treat various kinds of diseases, including insomnia and prosopoplegia. Moreover, clinical studies have found that insomniacs show more sensitivity to pinprick stimuli [15], so some noninvasive treatments like SA decrease tension and effectively improve sleep quality in chronic insomnia patients [16, 17]. Unfortunately, despite being an effective treatment for insomnia, the mechanism of SA is still unknown.

Recent studies showed that electroencephalograms (EEG) signals are time series and it could be used to assess changes in brain activity in real time [18] as EEG signals are highly complex, having nonlinear and nonstationary behavior [19]. Studies have also indicated that understanding brain dynamics in different time domains is through nonlinear dynamical analysis of EEG [20, 21], which is a branch of the emerging holistic science of complex systems [22]. In nonlinear dynamical systems, there is no relationship between independent and dependent variables, meaning that this system has the typical characteristics of coherence, inhomogeneity, and asymmetry [23]. Therefore, in this study, the Approximate Entropy (ApEn) and Correlation Dimension (CD) were used to extract the feature of EEG signals. With good antinoise and anti-interference abilities, the ApEn requires only a short data point to estimate the random signals and then determine the characteristics of the whole signal [20, 24], while CD is also considered one of several key measures in chaotic time series analysis, which can assess the degree of similarity in the time series between one observation and others [25].

Furthermore, the “complexity” of a living system, such as a human brain, is not a single uniform process or property that is easily captured with a single variable [25, 26]. Using simple conventional statistical methods arbitrarily divides the whole brain into a number of independent components without taking into account the group structure problem among the brain regions. Thus, the statistical results and conclusions obtained from these studies cannot accurately describe the central effect of SA. Therefore, the use of a combination between nonlinear analysis and multivariate statistics EEG measures gave the advantages to investigate the changes in different brain regions over time.

The structure of this paper is as follows. In the “Methods” section, the clinical trial procedure of SA and the corresponding nonlinear analysis methods are introduced. In the “Results” section, the results and corresponding data analysis are presented. Finally, the “Discussion” section is given. Overall, in this study, using nonlinear dynamics and multivariate statistics, we studied the EEG of primary insomnia under SA stimulation to determine what the implications are on the brain. The results and the proposed method in our study will be useful in laying a foundation for further study on the mechanism through which SA affects brain function.

## 2. Methods

### 2.1. Design

Focused on these changes of brain regions strongly implicated in superficial acupuncture intervention, this study was designed to compare EEG signals before, during, and after SA stimulation at the Shangen acupoint for insomnia patients based on the fact that EEG signals are time series. Research subjects are 30 patients with primary insomnia from the Department of Sleep Medicine at the Rehabilitation Hospital affiliated with Fujian University of Traditional Chinese Medicine. Potential candidates for the study were screened and fully informed of the study. Eligible participants received SA intervention for 10 minutes, and EEG signals were collected for 10 minutes at each state, including prointervention (resting state), intervention (intervention state), and postintervention (postintervention state). The data were conducted using nonlinear dynamics (including Approximate Entropy (ApEn) and Correlation Dimension (CD)) and multivariate statistics.

This clinical trial was carried out in accordance with the Declaration of Helsinki and reviewed and approved by the Medical Ethics Committee Board of the rehabilitation hospital affiliated with Fujian University of Traditional Chinese Medicine, Fuzhou, Fujian, China (number: 2017KY-001-01). All of the participants need to sign the written informed consent form before trial. The whole experiment was operated and recorded by one experimenter.

### 2.2. Participants and Recruitment

#### 2.2.1. Inclusion Criteria

Patients meeting all of the following criteria were enrolled in the study:

1. Patient has a Pittsburgh Sleep Quality Index (PSQI) score ≥6 and an insomnia history ≥1 month, and symptoms of insomnia persisting ≥3 days per week.
2. Patient has an incubation period of falling asleep at night ≥30 minutes and a total sleep time ＜6 hours.
3. Patient meets The Chinese Classification and the Diagnose Criterion of Mental Disorder (CCMD-3) [27].
4. Patient meets the DSM-5 (Diagnostic and Statistical Manual of Mental Disorders, 5th Edition) diagnostic criteria for insomnia [28].
5. Patient stops taking sedatives and tranquilizers, antidepressants and psychotropic drugs, and other drugs affecting sleep EEG signals.
6. Patient has no history of head trauma, epilepsy, head metal implantation, or pacemaker implantation.
7. Male and female patients between the ages of 18 and 75 years (inclusive) were eligible for participation.
8. Patient provides signed informed consent.

#### 2.2.2. Exclusion Criteria

Patients meeting any of the following criteria were excluded from the study:

1. Patient suffers from physical or mental disorders that result in progressive insomnia or other types of sleep disorders.
2. Patient works shifts involving changes in day/night work schedule that impacts circadian rhythm.
3. Patient is pregnant, breast-feeding, or preparing to become pregnant.
4. Patient has severe respiratory insufficiency and/or is given sedatives for 1 week.
5. Patient has cardiovascular, endocrine, viscera, and/or hematopoietic diseases.
6. Patient is an alcoholic or psychotropic substance abuser.
7. Patient is noncompliant with the treatment or study protocol.

#### 2.2.3. Withdrawal or Dropout Criteria

1. The participant has an adverse event caused by this study
2. The participant requires to drop out.

**Figure 2:**
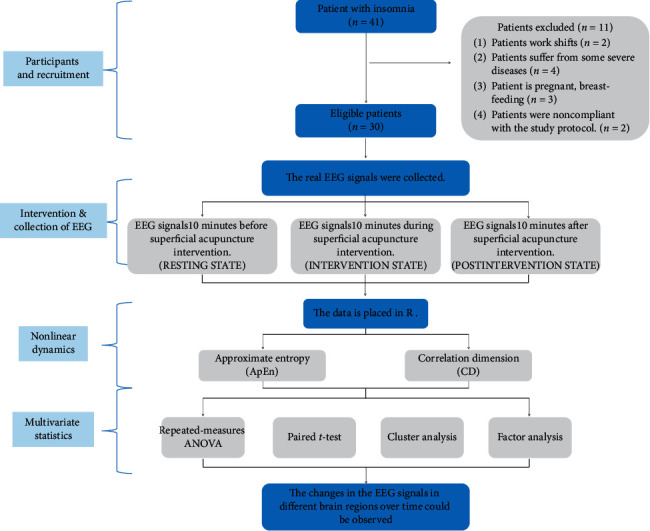
Flow chart.

We included 30 patients with insomnia in the study following the inclusion and exclusion criteria, all of whom were right-handed. There were 16 female cases and 14 male cases, between 28 and 73 years old. The flow diagram was performed in Figure 2.

### 2.3. Intervention

All of the SA treatments were completed by one acupuncturist. A piece of cotton was wrapped around the needle tip in order to reduce pain caused by the needle tip contacting the skin. Then it is gently put on the Shangen acupoint, which was selected based on the theory of acupuncture and traditional Chinese medicine (TCM), according to volume 1 of Dong Yi Bao Jian (Dongui Bogam). This acupoint is located on the midpoint between the bilateral inner canthus. As previously described, the acupuncturist held the needle handle with two right-hand fingers (index finger and middle finger)，and scraped the needle handle at a uniform velocity for 10 minutes with his or her thumb (see Figure 1(b)).

### 2.4. EEG Recording and Preprocessing

**Figure 3:**
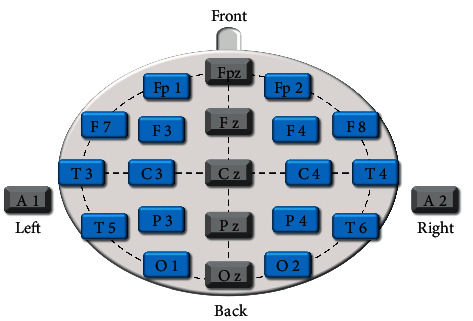
Schematic drawing of electrode positions.

This trial was conducted in a quiet, shielded room. The participants could not hear or see any distractions. EEG electrodes were placed according to the 10–20 international standard lead system (see Figure 3). The EEG signals were recorded using a 16-channel EEG from Beijing Upson Industry and Trade co., LTD. The data were acquired with the ZN16E high-frequency EEG signal amplifier, a pass-band filter of 0.3–100 Hz, and a mode/digital conversion digit of 12 bits. The sampling rate was 128 Hz (sampling interval 0.07 s). All electrodes were referenced to the earlobes, and 11000 data points were selected for each electrode.

When the instrument was in position, the subjects were asked to rest quietly with their eyes closed for 10 min before the data collection.

1. Each subject collected 10 min of EEG data before SA intervention (resting state)
2. Each subject collected 10 min of EEG data during SA intervention at Shangen acupoint (intervention state)
3. Each subject collected 10 min of EEG data after SA intervention (postintervention state)

### 2.5. Data Processing

The data processing was performed using the *R* programming language (version 3.6.3). As an open-source statistical language and a data analysis tool, *R* was first developed in 1993 by Ross Ihaka and Robert Gentleman at the University of Auckland [29], which is an interpretive language for statistics and graphics with efficient data processing and storage abilities [30].

Approximate Entropy (ApEn), introduced by Pincus [31, 32], is a measure of data regularity, that is, the conditional probability of similarity vector which maintains its similarity when it increases from *m* to *m* + 1 dimension. Its physical meaning is the size of the probability of generating a new pattern in a time series as the dimension changes ApEn measures the volatile trend of the research object, which is determined by the complexity of time series. The more complex the time series is, the higher the probability of generating new patterns will be and the larger the corresponding ApEn; this also reflects a higher degree of brain activity in terms of EEG [23, 33]. The formula is defined as follows:

(1) $$\begin{array}{c} \text{ApEn}\left(m,r\right)=\underset{n⟶\infty }{\text{lim}}\left[\varphi^{m}\left(r\right)-\varphi^{m+1}\left(r\right)\right]. \end{array}$$

In this study, one ApEn (*m* = 2, *R* = 0.2 sd) is calculated from every 100 data using the “pracma” command in *R*. Then, the mean values of ApEn are taken from each electrode.

In addition, Grassberger and Procaccia introduced CD, a geometric measure of dynamic complexity that can be used to estimate the size of phase space [34]. In terms of an EEG, the CD represents the degrees of freedom of human brain activity; the decreasing CD values indicate that the components of the brain are continuously coupled or that the previously active parts begin to become inactivated [19]. The formula is defined as follows:

(2) $$\begin{array}{c} \text{CD}=\underset{r⟶0}{\text{lim}}\left[\frac{\text{ln}C\left(r\right)}{\text{ln}r}\right]. \end{array}$$

Data were processed using *R* software, “nonlinear series” program.

### 2.6. Statistical Analysis

Data were analyzed using SPSS, version 22.0 (SPSS Inc). All statistical analyses were two-tailed tests and the level of significance was 0.05. Data were presented as mean values ± SD and 95% confidence intervals (CI) of the mean or frequencies. Measurements were analyzed by two-way repeated-measures ANOVA (time), paired *T*-test, cluster analysis, and factor analysis.

## 3. Results

### 3.1. Repeated-Measures ANOVA Results

**Figure 4:**
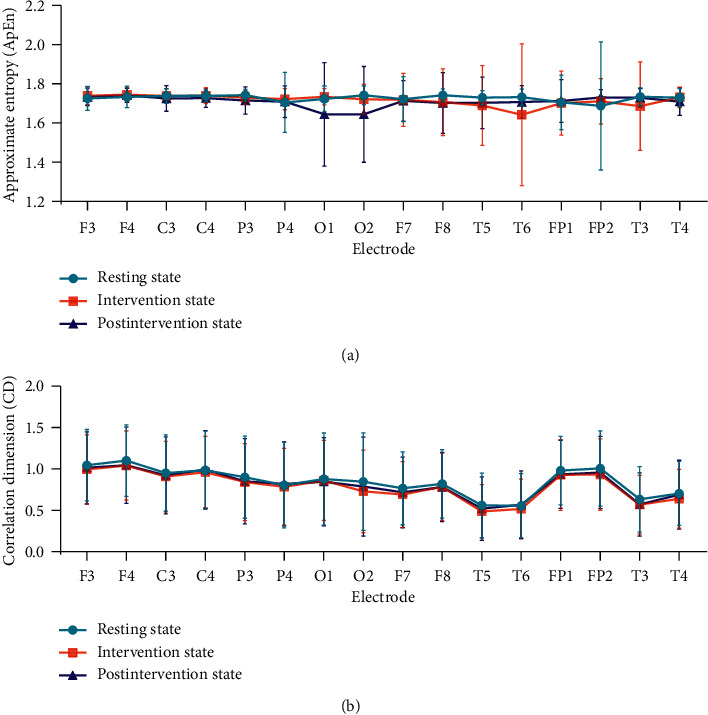
Repeated-measures ANOVA results.

 Repeated-measures ANOVA for ApEn (Figure 4(a)), *F* = 0.786, *p* = 0.776 ＞ 0.05 and repeated-measures ANOVA for CD (Figure 4(b)), *F* = 0.379, *p* = 0.999 ＞ 0.05.

### 3.2. Paired *T*-Test Results

The paired *t*-test results of ApEn and CD are shown in Table 1. The number with ^*∗*^ was the *p* value less than 0.05. That is, the ApEn values of electrodes O2 at the postintervention state decreased, compared with resting state (*p* < 0.05). The electrodes O2 were located in the right occipital lobe.

### 3.3. Cluster Analysis Results

**Figure 5:**
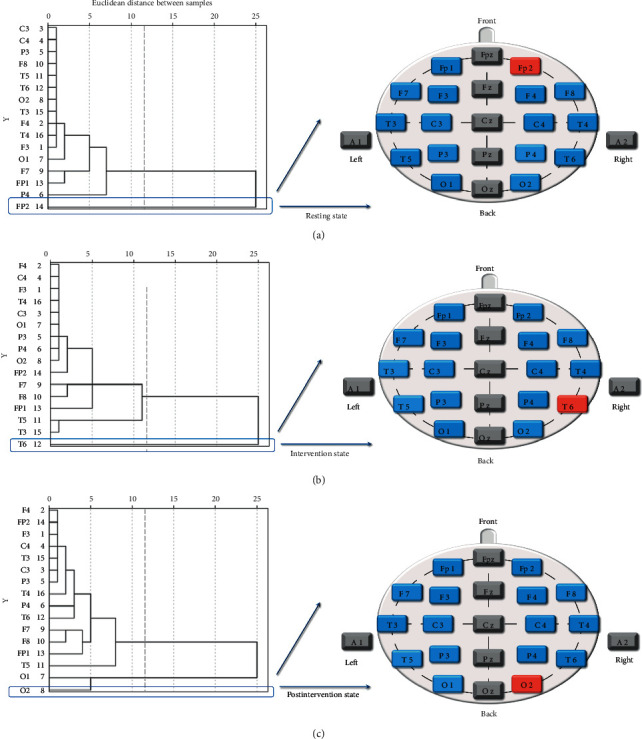
Cluster analysis diagram of ApEn at the three states. (a) Resting state, (b) intervention state (c) postintervention state.

**Figure 6:**
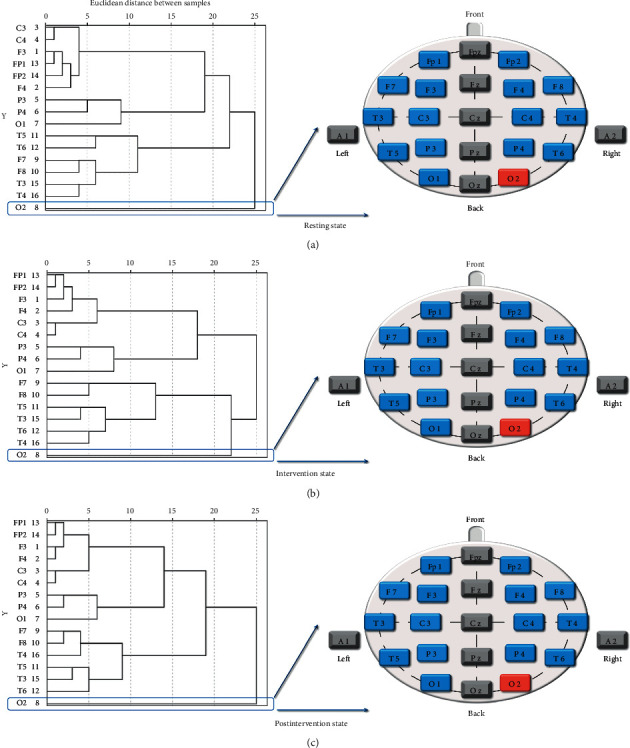
Cluster analysis diagram of CD at the three states. (a) Resting state, (b) intervention state, (c) postintervention state.

The dendrograms on the left of Figures 5 and 6 show the cluster analysis results of ApEn and CD. We extracted the cluster analysis results and drew them onto a schematic of electrode positions for visualization and better understanding, with blue representing normal control and red representing cluster analysis results.

The cluster analysis of ApEn revealed that the electrodes Fp2 at the resting state (Figure 5(a)), T6 at the intervention state (Figure 5(b)), and O2 at the postintervention state (Figure 5(c)) were the highest-ranking cluster. And the cluster analysis of CD indicated that the electrodes O2 at three states (Figure 6) was the only highest-ranking one.

### 3.4. Factor Analysis

**Figure 7:**
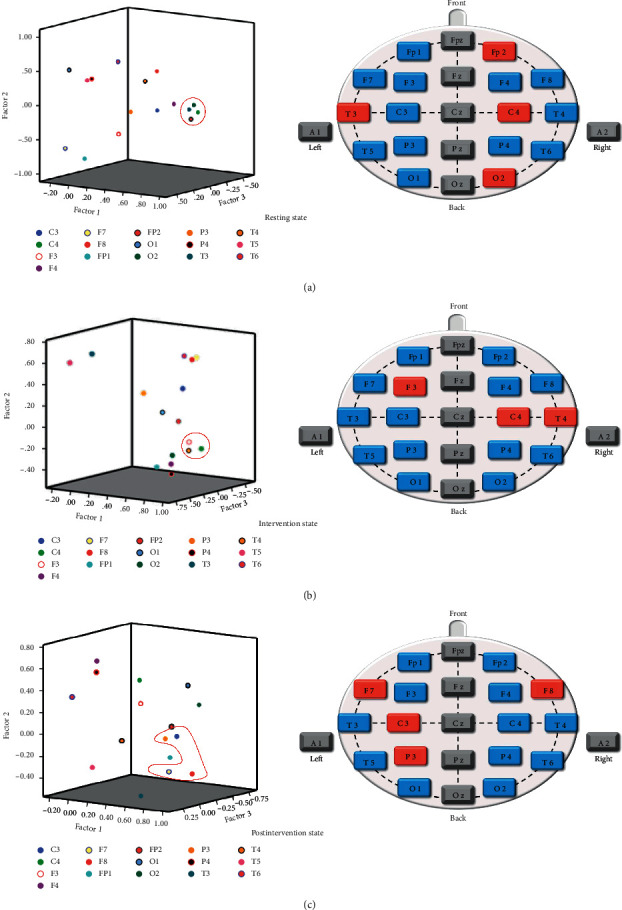
Factor analysis diagram of ApEn at the three states. (a) Resting state, (b) intervention state, (c) postintervention state.

**Figure 8:**
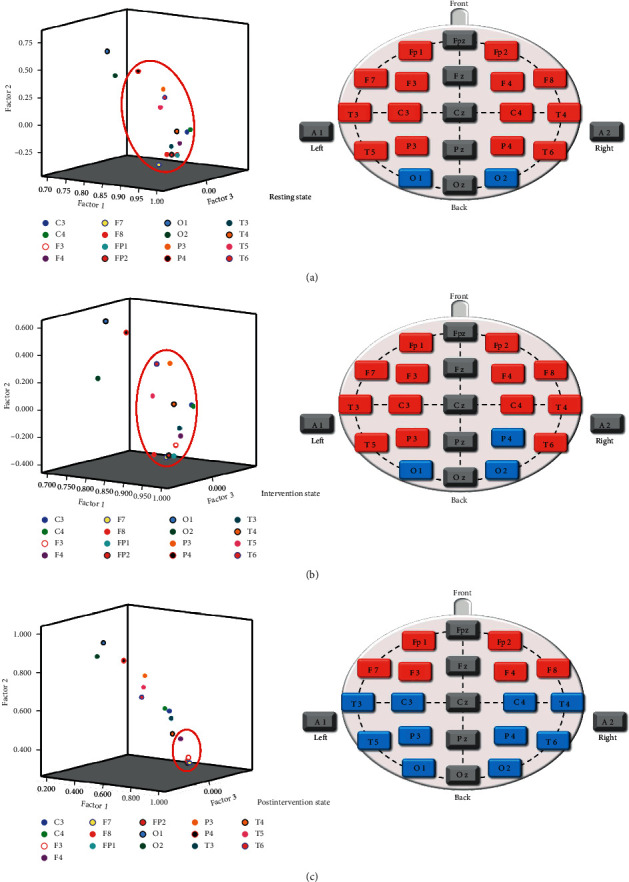
Factor analysis diagram of CD at the three states. (a) Resting state, (b) intervention state, (c) postintervention state.

Using the SPSS factor analysis procedure, we carried out a Principal Component Factor Analysis and selected the common factors which were cumulative contribution rate of the common factors ≥75% and the eigenvalues ≥1. The result diagrams on the left of Figures 7 and 8 show the factor analysis results of ApEn and CD.

Just as with the cluster analysis described above, we extracted the factor analysis results and drew them onto a schematic of electrode positions for visualization and better understanding, with blue representing normal control and red representing factor analysis results.

The factor analysis of ApEn revealed that the electrodes Fp2, T3, C4, O2 at the resting state (Figure 7(a)), F3, C4, T4 at the intervention state (Figure 7(b)), and F7, F8, C3, P3 at the postintervention state (Figure 7(c)) were more prominent.

And the factor analysis of CD indicated that the electrodes Fp1, Fp2, F3, F4, F7, F8, T3, T4, T5, T6, C3, C4, P3, and P4 at the resting state (Figure 8(a)), FP1, FP2, F3, F4, F7, F8, T3, T4, T5, T6, C3, C4, and P3 at the intervention state (Figure 8(b)), and Fp1, Fp2, F3, F4, F7, and F8 at the postintervention state (Figure 8(c)) were more prominent.

## 4. Discussion

Fundamental principles of metabolic coupling between different brain regions have recently been studied using neuroimaging techniques (fMRI, PET), proving that there is a specific relationship between acupoints and brain regions [35, 36]. This relationship is not a one-to-one correspondence between an acupoint and a single functional brain region, but multidimensional interactions between an acupoint and multiple distributed areas of the brain.

As the high spatial but low temporal correlation [37], the neuroimaging techniques could not detect significant changes in the connectivity of these neural networks structures under certain circumstance [19]. Some researches demonstrated that the temporal dynamics of an ongoing “stream of consciousness” is much faster than the time resolution of blood oxygen lever-dependent (BOLD) [38]. Therefore, in spite of its low spatial resolution, the EEG is more appropriate for obtaining the changes in different brain regions over time to understand the central mechanisms of SA intervention.

However, as the majority of physiological signals are by nature, nonstationary, and nonlinear, it is difficult to directly observe the effects of SA on the body over time using EEG [39]. There is no doubt that mathematics is integral to the study of biological systems that can be used to quantitatively explore and explain some phenomena better than compared to only experimental observations [29]. Therefore, this study put forward a method of combining the nonlinear dynamics with multivariate statistics to analyze the data. Nonlinear dynamics can serve as a fruitful tool that quantifies the complexity of a time series. Therefore, it can be used to evaluate the nonlinear, unstable EEG [32]. Overall, the multivariate statistics can provide a productive approach for detecting the overall changes and connectivity in brain regions after SA intervention.

The results of repeated-measures ANOVA in Figure 4 indicated no statistical difference both in ApEn and CD, suggesting that the use of nonlinear analysis gave the advantages to deal with the spectrum and complexity characteristics of the EEG signal and make it more consistent, synchronized, and stable. The cluster analysis results of ApEn were performed that the right prefrontal lobe (electrode Fp2) at the resting state was the highest-ranking one. That was consistent with other research. Some researchers found that compared to the control group, the beta1 power spectrum was lower in the prefrontal cortex in the insomnia group [40, 41]. Furthermore, we found that the SA stimulation affected the right posterior temporal lobe and then spread to the occipital lobe, and this result was in good agreement with the paired *t*-test results of ApEn, which showed the ApEn values of electrode O2 at postintervention state have decreased, compared with resting state (*p* < 0.05). The cluster analysis results of CD also showed the electrode O2 was the only highest-ranking one at three states. Some studies indicated that the mean occipital GABA/Cr ratio was significantly higher in the insomnia group than in the control group [42]. Therefore, we hypothesized that the cluster analysis of CD might highlight the abnormal EEG in the occipital lobe for insomniacs. In addition, the results of the factor analysis of CD performed that whole brain regions except the occipital lobes changed to the frontal poles, anterior temporal, and frontal lobes from pre-to postintervention.

**Figure 9:**
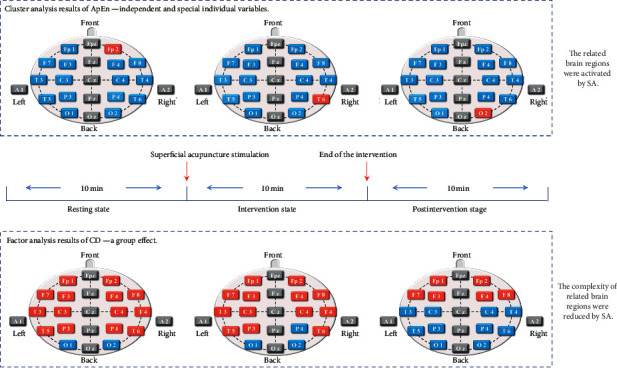
Schematic representation of the main findings from the study.

Cluster analysis classifies individuals into classes and identifies special variables within a cluster that are more similar to each other than the variables contained in different clusters [43]. It aims to delimit specially activated EEG signals. In other words, its purpose is to observe changes in EEG caused by SA stimulation. In addition, by retaining the original variable information and decomposing the original information to classify potential categories, factor analysis can determine which variables form coherent subsets (factors) that are somewhat independent and that can reflect the overall information [44, 45]. Therefore, unlike cluster analysis, using factor analysis approaches aims to assess the internal structure. That is to say, the results of cluster analysis were independent and special individual variables. The results of factor analysis were a group effect, meaning a global alteration of brain state (see Figure 9).

Moreover, ApEn and CD are each considered to reflect different aspects of data. ApEn is an algorithm used to measure the complexity of time series [20]. We found that the cluster analysis results of ApEn might be associated with brain activation by SA stimulation, reflecting needling-specific brain responses. And CD is one of the most basic quantitative indexes of chaotic time series used to measure the system of complexity by assessing attractor dimension in the reconstructed phase space [21], reflecting the correlation degree of EEG signals sequence itself [46]. Studies have shown that the magnitude of the CD reflected the frequency of activity of neurons in one particular brain region. The larger CD values, the more complex the neuron activity [47]. Therefore, based on the factor analysis results of CD, we hypothesized that one of the possible central mechanisms of SA intervention was the suppression of activity in the relevant brain regions, including the temporal, central, posterior temporal, and parietal lobes. It reduced the complexity of the brain regions involved. The obtained results were in good agreement with the medical report and MRI data, which demonstrated different insomnia-related heterotopic connectivity patterns in the right and left middle occipital/posterior middle temporal gyrus [48]. Besides, we have not found the regularity in factor analysis results of ApEn, nor in the cluster analysis of CD. It may be relevant that the ApEn is more suitable for cluster analysis, whereas CD is more suitable to factor analysis, according to their characteristics.

In summary, based on the fact that EEG signals are time series, it is feasible to use nonlinear dynamics analysis and multivariate statistics to examine the effects of SA on the human brain. In this study, the cluster analysis of ApEn may be the characterization of specially activated EEG signals by SA. That is, patients' EEG changed from the right prefrontal lobe to the right posterior temporal lobe and finally to the right occipital lobe, before, during, and after the SA intervention. While the factor analysis of CD may be the characterization of an overall alteration of brain state, showing that suppression of activity in the relevant brain regions, including the temporal, central, posterior temporal, and parietal lobes. We referred these changes to “the coupling effect among multiple brain regions.” It means taking the temporal and spatial factors as parameters, the characteristics and connectivity of the overall changes between brain regions after intervention are revealed. We hope to apply this method to more studies on the mechanism of SA and acupuncture in the future, and it provides a new way to study the temporal and spatial characteristics of the influence of acupuncture on the central nervous system.

## Figures and Tables

**Table 1:** Paired *t*-test results (mean ± SD, *n* = 30).

Mean ± SD						
Electrode	Approximate entropy (ApEn)			Correlation dimension (CD)		
	Resting state	Intervention state	Postintervention state	Resting state	Intervention state	Postintervention state
F3	1.73 ± 0.06	1.75 ± 0.05	1.74 ± 0.04	1.06 ± 0.43	1.01 ± 0.42	1.03 ± 0.44
F4	1.74 ± 0.05	1.75 ± 0.04	1.75 ± 0.04	1.12 ± 0.43	1.06 ± 0.42	1.06 ± 0.46
C3	1.75 ± 0.04	1.75 ± 0.04	1.73 ± 0.07	0.97 ± 0.46	0.93 ± 0.43	0.94 ± 0.46
C4	1.75 ± 0.03	1.75 ± 0.04	1.73 ± 0.05	1.00 ± 0.47	0.98 ± 0.43	1.01 ± 0.47
P3	1.75 ± 0.03	1.74 ± 0.03	1.72 ± 0.07	0.92 ± 0.50	0.86 ± 0.47	0.87 ± 0.52
P4	1.71 ± 0.15	1.73 ± 0.05	1.72 ± 0.08	0.82 ± 0.52	0.80 ± 0.46	0.84 ± 0.51
O1	1.73 ± 0.07	1.74 ± 0.04	1.65 ± 0.26	0.90 ± 0.56	0.88 ± 0.48	0.86 ± 0.53
O2	1.75 ± 0.05	1.73 ± 0.08	1.65 ± 0.24^*∗*^	0.87 ± 0.59	0.75 ± 0.50	0.81 ± 0.60
F7	1.73 ± 0.12	1.73 ± 0.14	1.72 ± 0.10	0.78 ± 0.44	0.71 ± 0.40	0.74 ± 0.43
F8	1.75 ± 0.03	1.71 ± 0.17	1.71 ± 0.16	0.84 ± 0.41	0.80 ± 0.41	0.80 ± 0.42
T5	1.74 ± 0.04	1.70 ± 0.20	1.71 ± 0.13	0.58 ± 0.39	0.51 ± 0.32	0.54 ± 0.38
T6	1.74 ± 0.04	1.65 ± 0.36	1.72 ± 0.08	0.57 ± 0.39	0.54 ± 0.36	0.89 ± 0.41
FP1	1.71 ± 0.14	1.71 ± 0.16	1.72 ± 0.11	1.00 ± 0.41	0.95 ± 0.43	0.95 ± 0.41
FP2	1.70 ± 0.33	1.72 ± 0.12	1.74 ± 0.04	1.02 ± 0.46	0.95 ± 0.43	0.97 ± 0.44
T3	1.74 ± 0.05	1.69 ± 0.23	1.74 ± 0.05	0.65 ± 0.40	0.59 ± 0.36	0.59 ± 0.38
T4	1.74 ± 0.05	1.74 ± 0.05	1.72 ± 0.07	0.72 ± 0.39	0.66 ± 0.35	0.71 ± 0.42

## Data Availability

The datasets used and/or analyzed during the current study are available from the corresponding author upon reasonable request.
